# Short-Term Adaptation of Joint Position Sense Occurs during and after Sustained Vibration of Antagonistic Muscle Pairs

**DOI:** 10.3389/fnhum.2014.00896

**Published:** 2014-11-04

**Authors:** Tomas I. Gonzales, Daniel J. Goble

**Affiliations:** ^1^School of Exercise and Nutritional Sciences, San Diego State University, San Diego, CA, USA; ^2^Motor Control Laboratory, School of Kinesiology, University of Michigan, Ann Arbor, MI, USA

**Keywords:** proprioception, vibration, adaptation, upper-limb, muscle spindle, kinesthesia

## Abstract

Proprioception is critical for the control of many goal-directed activities of daily living. While contributions from skin and joint receptors exist, the muscle spindle is thought to play an important role in allowing accurate judgments of limb position and movement to occur. The discharges elicited from muscle spindles can be degraded by simultaneous agonist-antagonist tendon vibration, causing proprioception to be distorted. Despite this, changes in limb perception that may result from sensory adaptation to this stimulus remain misunderstood. The purpose of this study was, therefore, to investigate short-term proprioceptive adaptation resulting from vibration of antagonistic muscle pairs. We measured elbow joint position sense in 21 healthy young adults while 80 Hz vibration was applied simultaneously to the distal tendons of the elbow flexor and extensor muscles. Matching errors were then analyzed during early and late adaptation phases to assess short-term adaptation to the vibration stimuli. Participants committed significant undershoot errors during the early adaptation phase, but were comparable to baseline measurements during the late adaptation phase. When we removed the vibration stimuli and conducted a second joint position matching task, matching variability increased significantly, and participants committed overshoot errors. These results bring into question the efficacy of simultaneous agonist-antagonist tendon vibration to degrade proprioceptive acuity.

## Introduction

Sensory adaptation is a term that relates to the ability of a sensory system to change responsiveness over time. This process can be predictive in nature, as might be necessary for the case of sensory-motor learning. Alternatively, sensory adaptation can be reactive, serving as a means for allowing equilibrium states to be achieved in the face of external stimuli. In this way, the time course of sensory adaptation is both situation dependent, and dependent on the strength and duration of sensory stimuli.

While a multitude of studies have investigated sensory adaptation of the traditional five senses (i.e., sight, sound, smell, taste, and touch), few attempts have been made to specifically explore this phenomenon in the proprioceptive system (Desmurget et al., 2000; Seizova-Cajic et al., 2007). Proprioception is a term first coined by Sherrington (1907) that referred to the set of bodily sensations generated during one’s own actions. Over time, the term has come to be defined as the means by which an individual is able to sense and perceive body positions in the absence of vision (for review, see Proske and Gandevia, 2009). Regardless, it has been shown that the underlying neural signals that subserve proprioceptive sense arise from joint, cutaneous, and muscle spindle receptors. Of these “proprioceptors,” it is feedback from muscle spindles that is thought to play a particularly pivotal role in allowing accurate judgments of limb position and movement to be made (Burke et al., 1976; Roll and Vedel, 1982; Roll et al., 1989).

The importance of muscle spindle signals for proprioceptive sense has most clearly been demonstrated through tendon vibration studies. This experimental paradigm encompasses the application of a mechanical vibration stimulus to the tendon of a target muscle in order to stimulate primary (Ia) muscle spindles. It has been shown using microneurography that the rate of muscle spindle firing increases harmonically in response to tendon vibration (Roll and Vedel, 1982), and that the increased neural signal is perceived as lengthening of the muscle by the brain (Goodwin et al., 1972). Interestingly, this illusory response decreases with prolonged stimulation, and cessation of the vibratory stimulus elicits a transitory (i.e., 30 s) kinesthetic aftereffect in which the vibrated limb seems to be moving in the opposite direction of the illusory movement (Seizova-Cajic et al., 2007). This response is believed to correspond to a depression in the firing rate of muscle spindle primary afferents (Ribot-Ciscar et al., 1998), although adaptation at other locations within the nervous system is likely.

A great deal of evidence now exists supporting the notion that perception of joint movement is based on the central appreciation of primary muscle afferent activity originating from both the shortening and lengthening muscles. More specifically, an imbalance of inputs from agonist and antagonist muscles results in the perception of motion in the corresponding direction (Gilhodes et al., 1986). During natural voluntary movements, this imbalance favors the lengthening muscle, since primary muscle afferents will typically fail to awaken in the shortening (i.e., agonist) muscle (Roll and Vedel, 1982). Mechanical vibration can, therefore, be used to confound information from these channels of proprioceptive information by interfering with the ability of muscle spindles to respond to naturally evoked stimuli (Roll et al., 1989).

In light of the above findings, several recent attempts have been made to demonstrate the applicability of simultaneous agonist-antagonist mechanical tendon vibration to degrade proprioception in healthy adults (Bock et al., 2007; Vidoni and Boyd, 2008; Ronsse et al., 2009). This dual agonist-antagonist vibration approach might serve as an important research technique, as it could provide a feasible and reversible means for studying the consequences of poor proprioception, known to be characteristic of numerous clinical populations (Smith et al., 1983; Sainburg et al., 1993; Goble et al., 2012). It is yet unknown, however, to what extent the nervous system adapts to mechanical tendon vibration when applied to both agonist and antagonist muscle pairs. This adaptation processes, presumably mediated through changes in muscle spindle activity, may parallel processes observed in other types of sensory receptors following sustained vibratory stimulation. For example, cutaneous mechanoreceptors become desensitized to suprathershold vibration after prolonged periods of stimulation (Bensmaia et al., 2005) and the time-course of adaptation is faster than perceptual measures observed during psychophysical experimentation (Leung et al., 2005). It is reasonable to predict that muscle spindle adaptation processes may be similar to those observed during cutaneous mechanoreceptor stimulation. If so, this would bring into question whether dual agonist-antagonist tendon vibration may be used in lieu of other known reversible methods to reduce proprioceptive acuity, such as ischemic nerve block.

The aim of the present study was, therefore, to determine whether short-term (i.e., within 10 trials) proprioceptive adaptation occurs in response to simultaneous agonist-antagonist tendon vibration. This was accomplished by measuring proprioceptive bias (i.e., constant error) and variability (i.e., variable error) in an elbow joint position matching task before, during, and after continuous dual vibration of the biceps and triceps muscle tendons for approximately 10 min. Proprioceptive bias and variability were analyzed during early and late periods within each phase of the matching experiment to assess perceptual changes to the dual vibration stimuli over time. We hypothesized that only matching variability would increase during the early period of the dual vibration phase of the experiment and diminish during the late period due to proprioceptive adaptation. Alternatively, based on previous work examining changes in proprioceptive bias following dual vibration (Ronsse et al., 2009), it could have been hypothesized that constant error (i.e., bias) would also change appreciably when the vibration stimuli were applied and removed. In this case, adaptation to the perturbation would be expected such that bias was reduced from early to late matching trials.

## Materials and Methods

### Participants

Informed consent was obtained from a convenience sample of 21 healthy young adults (12 males; 9 females; mean ± SD, age = 26.6 ± 4.6 years) prior to their participation in the study. Exclusion criteria for participants were any self-reported history of upper-limb sensorimotor deficits or cognitive impairment, as well as any tendency toward left handedness measured using the Edinburgh Handedness Inventory (Oldfield, 1971). Procedures for this study were approved the institutional review board at San Diego State University.

### Experimental setup

For all elbow position matching trials (see Experimental Procedure below) participants were seated in a height adjustable chair with their dominant, right forearm resting in a padded cast on a horizontally rotating robotic manipulandum shaft driven by a programmable torque motor (Kollmorgen servomotors, AKM13D 7000RPM @ 160VDC). The height of the chair was adjusted so that the manipulandum shaft was at the level of the xiphoid process. The axis of rotation of the elbow was aligned with the rotational axis of the manipulandum and elbow angle data were digitized and processed using custom software developed in the LabVIEW environment (National Instruments, TX, USA). To minimize the influence of sensory information from the left arm on right elbow proprioception (Izumizaki et al., 2010), the left arm was positioned comfortably on the participant’s lap and was not moved during testing. Participants were randomly assigned to two experimental groups (FLEXED or EXTENDED) to counterbalance any differences due to movement of the arm toward the proprioceptive targets. The starting posture of the elbow to be tested was 90° of flexion in the FLEXED group and 0° flexion (i.e., full extension) in the EXTENDED group.

The triceps and biceps brachii muscles of the testing arm were fitted with cylindrical electromechanical vibrators secured using an elastic arm sleeve (Nike Men’s Arm Sleeve). Specifically, the biceps brachii tendon vibrator was positioned perpendicular to biceps tendon about 1 cm proximally from the cubital fossa and the triceps brachii tendon vibrator was positioned perpendicular to the distal triceps tendon about 2 cm proximally from the olecranon. The vibrators were calibrated to stimulate the muscle spindles at 80 Hz with amplitude of ~1 mm. In agreement with previous work, participants reported no proprioceptive illusions when both vibrators operated simultaneously at this frequency (Gilhodes et al., 1986). All participants were blindfolded and wore noise canceling headphones during testing, which respectively served to eliminate any visual and/or auditory feedback regarding movement or position of the elbow joint.

### Experimental procedure

Each testing session consisted of three experimental conditions under which proprioceptive matching of elbow joint angles was performed. The first condition was a baseline condition (BASE) where participants completed proprioceptive matching with the vibrators turned off to obtain pre-vibration levels of proprioceptive bias and variability. The second condition (VIB) consisted of proprioceptive matching while adapting to vibration applied to the biceps and triceps tendons simultaneously. The last condition (AFTER) was identical to the first condition (i.e., BASE), and was conducted to assess de-adaptation following the VIB condition.

**Figure 1:**
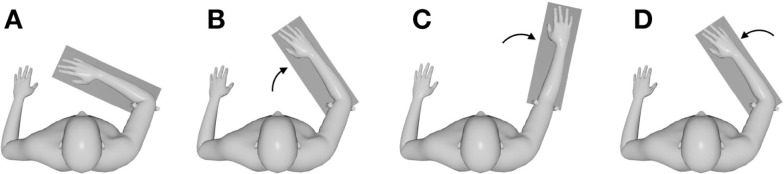
**Top-down perspective of the experimental setup and protocol in the FLEXED group**. Position of the forearm initially **(A)**, when moving toward and being held stationary at the angular target **(B)**, when moving and being held stationary at an extended posture **(C)**, and when returning to the initial position to match the angular target **(D)**. The shaded rectangle represents the position of the manipulandum throughout testing.

Elbow matching was performed according to the following protocol for all conditions (illustrated in Figure 1). Prior to testing, participants were instructed to completely relax their arm, to not move throughout testing, and to not interfere with any movements imposed by the manipulandum. The manipulandum was programed to shut off when any low level resistance was detected. Once participants were relaxed, the forearm was moved by the manipulandum to an angular target between 35 and 55° from the initial elbow angle. This target was in the direction of extension for the FLEXED group and in the direction of flexion for the EXTENDED group. When the angular target was reached, the manipulandum stopped moving and the forearm was held stationary for 3 s while the participant memorized the location based on proprioceptive information. Next, the arm was moved by the manipulandum to 0° of flexion in the FLEXED group and 90° of flexion in the EXTENDED group. The forearm was held stationary at this position for 3 s and then was returned to the initial position. While the forearm was being returned, participants indicated when the memorized angular target was achieved by pressing a mouse button with their contralateral hand. Participants were instructed to press the mouse button when they perceived their elbow angle to be equal to the target position. All arm movements were passive and had a constant velocity of 5°/s. Each trial was approximately 40 s in duration, and vibratory stimulus was sustained throughout the duration of all vibration trials (~10 min). The duration of the vibratory stimulus aligns with previous work indicating that simultaneous agonist-antagonist vibration increases position uncertainty after 20 s of sustained vibratory stimulus (Fuentes et al., 2012).

Five angular targets were used for testing trials: 35, 40, 45, 50, and 55° from the starting position of the elbow. Angular targets with the same amplitude were not presented subsequently in order to prevent their memorization across trials. The inter-trial delay was 6–8 s as randomly specified by the testing computer, and the three experimental conditions were presented concurrently with no appreciable delay between conditions. An early adaptation and late adaptation phase, each consisting of five trials, were conducted consecutively for the VIB and AFTER conditions in order to assess short-term adaptation/de-adaptation to the vibration stimuli. Trials were grouped in this manner to account for the effects of different movement amplitudes on position sense errors (Goble, 2010).

### Data analysis

Constant error and variable error were used to determine proprioceptive accuracy. Constant error is a measure of proprioceptive bias (i.e., underestimation or overestimation of angular targets) and was calculated by subtracting the matching angle from the target angle in degrees. Negative constant error values indicated undershooting of angular targets and positive values indicated overshooting. Variable error is an accepted measure of trial to trial variability for proprioceptive matching tasks and was determined as the standard deviation of constant error trials across each block in each subject. Higher variable error measures are indicative of increased position uncertainty (Goble, 2010).

### Statistical analysis

Constant error and variable error measures obtained during the VIB and AFTER conditions were normalized to mean performance during the BASE condition. Mean constant error was calculated for trials conducted during the BASE condition for each participant. This value was subtracted from mean constant error values obtained during the early and late adaptation phases of the VIB and AFTER conditions. The same procedure was used to normalize variable error. Proprioceptive performance was analyzed in this manner to determine the degree of sensory adaptation across each condition, irrespective of performance during the BASE condition.

Multiple one-sample *t*-tests were conducted to determine whether constant error and variable error during the early and late adaptation phases of the VIB and AFTER conditions were statistically significantly different from the BASE condition. In all, 95% confidence intervals were calculated for each comparison, and effect sizes were computed as Cohen’s *d*. Differences were considered significant with respect to an alpha of *p* < 0.05.

## Results

### Changes in proprioceptive bias (i.e., constant error)

**Figure 2:**
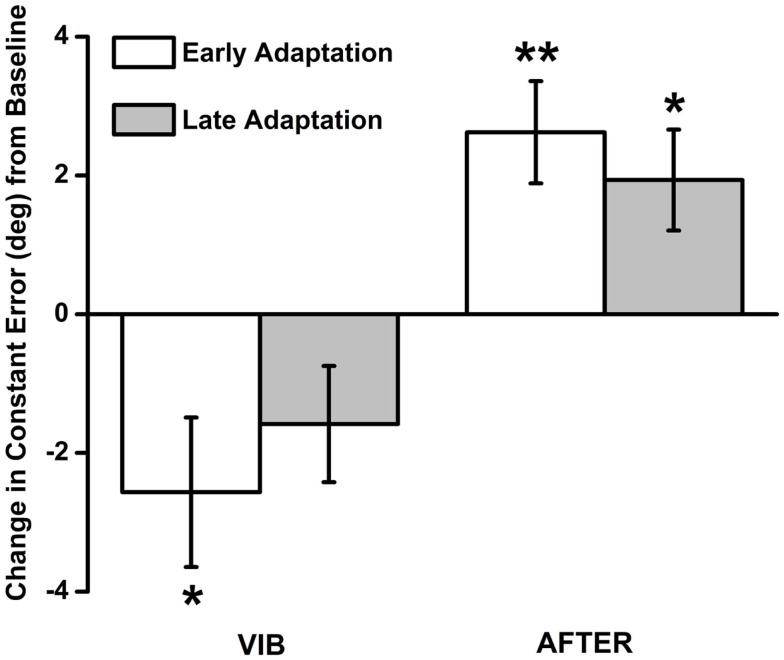
**Constant error results during the VIB and AFTER conditions**. Significant undershoot errors were observed during the early adaptation phase of the VIB condition. When the vibration stimuli were removed, participants committed overshoot errors during both early and late adaptation phases.

As expected, no participant reported having an illusion of movement about the elbow joint. Despite this, as shown in Figure 2, participants had greater undershooting during the early VIB adaptation phase, as shown by negative constant errors that were statistically significantly lower than BASE measures of proprioceptive bias [*t*(20) = −2.383, *p* = 0.027, 95% CI: −4.81, −0.32, *d* = −1.07]. Mean constant error during the late VIB phase (mean = −1.58 ± 0.84) was not statistically significantly different from BASE [*t*(20) = −1.89, *p* > 0.05, 95% CI: −3.33, 0.16, *d* = −0.85]. When vibration was removed, mean constant error during the early AFTER phase (mean = 2.62 ± 0.74) was statistically significantly greater than BASE measures [*t*(20) = 3.56, *p* = 0.02, 95% CI: 1.09, 4.16, *d* = 1.59], indicating greater overshooting. Mean constant error during the late AFTER phase (mean = 1.93 ± 0.73) was also statistically significantly greater [*t*(20) = 2.66, *p* = 0.15, 95% CI: 0.42, 3.45, *d* = 1.19], although the size of difference was closer to baseline.

### Changes in matching variability (i.e., variable error)

**Figure 3:**
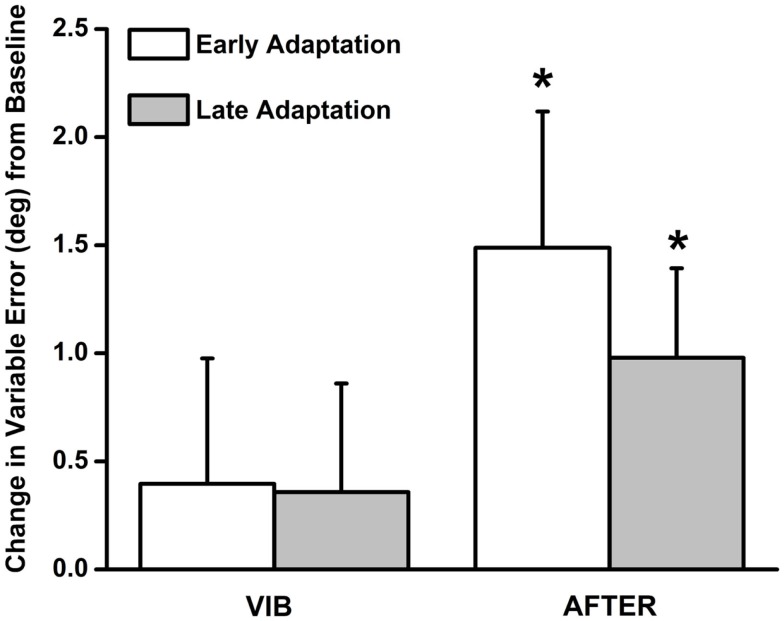
**Variable error results during the VIB and AFTER conditions**. No appreciable increase in variable error was observed during the VIB condition. In contrast, matching variability was significantly elevated following removal of the vibration stimuli. The magnitude of variable errors diminished from early to late adaptation phases in the AFTER condition.

Variable error (mean = 0.40 ± 0.58), shown in Figure 3, was not statistically significantly different from baseline when vibration was applied during both the early VIB [*t*(20) = 0.68, *p* > 0.05, 95% CI: −0.81, 1.61, *d* = 0.31] and late VIB (mean = 0.36 ± 0.50) [*t*(20) = 0.71, *p* > 0.05, 95% CI: −0.69, 1.41, *d* = 0.32] phases. Interestingly, when vibration was removed, variable error (mean = 1.49 ± 0.63) increased significantly during the early AFTER phase [*t*(20) = 2.36, *p* = 0.029, 95% CI: 0.17, 2.80, *d* = 1.05]. Variable error (mean = 0.98 ± 0.41) remained statistically significantly greater than baseline [*t*(20) = 2.36, *p* = 0.028, 95% CI: 0.12, 1.84, *d* = 1.06] in the late AFTER phase, and was closer in magnitude to baseline measurements.

## Discussion

Our sense of limb position and movement (i.e., proprioception) depends on afferent information conveyed to the central nervous system by muscle spindles, known to be sensitive to tendon vibration (Burke et al., 1976; Roll et al., 1989). Several investigations suggest that vibration degrades the quality of this afferent information, causing joint proprioception to be disrupted (Roll et al., 1989; Bock et al., 2007). To investigate how the proprioceptive system adapts in the short-term to perceptual perturbations caused by vibration, we conducted an elbow joint position matching task on healthy young adults while applying vibration to the distal tendons of the biceps and triceps brachii muscles. The vibration stimuli caused participants to commit undershoot errors when replicating reference joint angles. The magnitude of undershoot errors decreased over the course of the task. Following completion of the task, we removed the vibration stimuli and conducted a second joint position matching task. Matching performance on the second matching task revealed an aftereffect consisting of overshoot errors and a significant increase in matching variability.

Increased matching variability immediately following the removal of the vibration stimuli, but not when the stimuli were being applied, may be best interpreted in light of known changes to muscle spindles following prolonged vibratory stimulation. Ribot-Ciscar et al. (1998) investigated postvibration effects on the firing properties of a small population of muscle spindles located in the ankle dorsiflexor muscles. The spontaneous firing rate of most muscle spindles in the population decreased, while the rate of a subpopulation increased. Additionally, when the ankle was passively stretched, the mean firing rate of the spindle population was diminished and highly variable compared to previbratory measurements. These results parallel those of the present study, where the increase in matching variability that we observed was likely due to an increase in the variability of the spindle population’s response to stretch. Changes in the variability of firing properties of muscle spindles, however, may be dependent on the duration of the proceeding vibration stimuli. Fuentes et al. (2012) found that variability in the perceived angular position of the wrist joint increased significantly only after 20 s of simultaneous agonist-antagonist tendon vibration. The VIB phase of the present study consisted of ten trials, with each trial lasting approximately 40 s. This effect, therefore, appears to only emerge after prolonged periods of simultaneous vibration. Collectively, these results provide additional evidence for the theory that sensations of limb position and movement depend on responses from entire spindle populations (i.e., population coding) (Ribot-Ciscar and Roll, 1998; Cordo et al., 2002).

In light of the observed increase in matching variability following vibration, we hypothesize that the central nervous system cannot integrate proprioceptive information proficiently when the neuronal variability of the muscle spindle population exceeds a certain threshold level. We define this threshold as the amount of neuronal variability that will cause the population to have a widely spread response distribution. Our hypothesis is not unfounded given the roll of the fusimotor system in controlling the stretch sensitivity of muscle spindles (Hulliger, 1984). Further, the proposed hypothesis is supported by our observation that matching variability did not increase when the vibration stimuli were applied. Previous work has demonstrated that muscle spindles respond harmonically to vibratory stimulus that is within the 80–120 Hz range (Roll and Vedel, 1982). Since the vibrators in the present study operated invariantly at this frequency, it is unlikely that the vibration stimuli were capable of increasing the variability of spindle responses beyond the proposed threshold. Rather, vibrators designed to operate stochastically with sufficient amplitude may achieve this aim. Investigating this would require elements from information theory and is beyond the scope of this manuscript.

Simultaneous vibration of agonist and antagonist muscles caused participants to commit undershoot errors when replicating joint angles. Similar alterations of proprioceptive bias have been revealed when agonist or antagonist muscle groups are vibrated alone (Capaday and Cooke, 1981, 1983; Cordo et al., 1995). Since we vibrated the biceps and triceps brachii simultaneously, how might these previous investigations corroborate with the results of the present study? Illusions of joint movement can be elicited if the vibration frequency applied to antagonist muscle pairs is different (Gilhodes et al., 1986). The perceived velocity of these illusory movements is proportional to the difference in frequency between vibrators (Roll and Vedel, 1982; Ribot-Ciscar and Roll, 1998). If the same vibration frequency is applied, vibratory afferent information from the opposing muscle groups is negated when integrated by the central nervous system. Therefore, one would expect that agonist-antagonist muscle vibration would cause no appreciable change in joint position bias.

The changes in proprioceptive bias we observed in the present study may be accounted for by the joint position matching task that we used. We passively rotated the elbow in opposite directions when the reference angle was presented and reproduced. This caused participants to rely on different sources of afferent information when memorizing and matching reference angles. For example, in the FLEXED condition, the reference angle was memorized using afferent feedback from the elbow flexors and reproduced using elbow extensor feedback. The weighting of afferent information from each group of muscle may have been different, resulting in changes in proprioceptive bias when the muscles were vibrated (Mel’nichouk et al., 2007). Also, passive joint rotations reduced the effects of alpha-gamma coactivation on intrafusal fiber slack. This intrafusal slack could cause the firing properties of muscle spindles in the shortening muscles to be dependent on the history of the previously imposed stretch (Proske et al., 1992; Kostyukov and Cherkassky, 1997).

The magnitude of undershooting errors decreased over the duration of the vibration stimuli. Similarly, participants committed overshoot errors when the vibration stimuli were removed. These findings corroborate with results obtained by Seizova-Cajic et al. (2007) who found that movement illusions wavered when vibratory stimulation was applied to the elbow flexors over an extended period of time. Overshoot errors following vibration have been reported previously as well (Rogers et al., 1985; Gregory et al., 1988). Collectively, these results suggest that the proprioceptive system adapts to sustained afferent feedback caused by tendon vibration. The purpose of proprioceptive adaptation under natural conditions is likely analogous to other sensory systems: to maintain the sensitivity of the sensory system to changes in the surrounding environment (Helson, 1948). We propose that proprioceptive adaptation ensures that the central nervous system is capable of perceiving changes in limb position and movement when proprioceptors are continuously activated by self-generated movements. Supraspinal (Ebner and Pasalar, 2008; Mulliken et al., 2008) and intraspinal (Hantman and Jessell, 2010) mechanisms involved in the integration of corollary and sensory feedback likely play a crucial role in allowing the proprioceptive system to adapt over time.

There are several limitations in the present study worthy of recognition. First, we did not use electromyography to monitor activity of the biceps and triceps brachii during testing. We therefore can only speculate as to whether participants were able to completely relax. Further, since muscle activity was not monitored, we cannot discount the possibility that the tonic vibration reflex may have influenced our findings. Considerable effort was made to ensure that participants remained relaxed throughout the testing period, and the manipulandum was programed to operate only when it detected minimal resistance to imposed movements. Other sensory receptors, such as cutaneous mechanoreceptors, may have been influenced by their respective adaptive processes (Bensmaia et al., 2005; Leung et al., 2005). Therefore, this study does not explicitly describe the time-course of adaptation in muscle spindles following vibration. Rather, it provides evidence for adaptation processes that may occur throughout the proprioceptive system.

In conclusion, the results of the present study bring into question the use of dual agonist-antagonist tendon vibration to degrade proprioception about an adjacent joint. While previous investigations have used vibration to temporarily degrade proprioceptive feedback (Bock et al., 2007; Vidoni and Boyd, 2008; Ronsse et al., 2009), our results suggest this method elicits changes in proprioceptive bias that diminish with time and has little effect on variable error, as would be expected in the case of increased proprioceptive noise. Rather, removal of the vibration stimulus caused the most powerful increase in limb position uncertainty. We hypothesize that this may be due to limitations in the capacity of the central nervous system to integrate sensory input that is predominantly stochastic. Future investigations of the time-course underlying increases in limb position uncertainty following sustained tendon vibration are warranted.

## Conflict of Interest Statement

The authors declare that the research was conducted in the absence of any commercial or financial relationships that could be construed as a potential conflict of interest.
